# Editorial: Insights in cancer pain: 2022

**DOI:** 10.3389/fpain.2023.1236538

**Published:** 2023-07-13

**Authors:** Akiko Okifuji

**Affiliations:** Department of Anesthesiology, School of Medicine, The University of Utah, Salt Lake City, UT, United States

**Keywords:** cancer pain, bone cancer, chemotherapy-induced neuropathic pain, breakthrough pain, acupuncture

**Editorial on the Research Topic** Insights in cancer pain: 2022

Cancer is not a single disease entity, and the etiology, pathophysiology, course, treatment, and prognosis vary greatly across different types of cancer. Significant advancement in biomedical and biotechnological science in recent decades has contributed to the remarkable proliferation of life-saving treatments for many forms of cancer. Nevertheless, cancer remains to be one of the most feared disease (1). The major reason behind this fear is pain and associated functional impairment (2, 3). Indeed, cancer pain is prevalent and significantly impairs the sense of well-being and quality of life for patients (4). In light of this continuing challenge, we are especially pleased to have this issue that focuses on cutting-edge perspectives on cancer pain. The issue contains four excellent papers, two focusing on the mechanistic aspects of cancer pain and the other two focusing on therapeutic modalities.

A concise review by Chen et al. provides an excellent overview of the role of TRPV1 in dorsal root ganglion in pain associated with bone cancer. Given that moderate to severe pain is experienced by the majority of patients with bone cancer (5), a better understanding of the modulatory influence of the immune system and endogenous formaldehyde may be a game-changer and help us delineate specific therapeutic targets to treat bone cancer pain.

Johnson et al. provide a comprehensive review of genetic variations as a biomarker of chemotherapy-induced neuropathic pain (CINP). CINP is common iatrogenic response to chemotherapy for breast cancer patients, yet the presence and severity of CINP vary greatly across individuals. Factors that lead to the development of CINP are poorly understood. What we do know is that CINP is difficult to treat and adversely impacts patients' QOL, often leading to dose reduction or limitation of potentially life-saving chemotherapy (6). Johnson and his group provide an outstanding narrative review of the current understanding of genetic biomarkers for paclitaxel-induced neuropathy. Understanding how genetic variations and epigenetic changes could lead to the development of precision medicine algorithms to prevent and manage CINP.

Bossi et al. provide a brilliant overview of rapid-onset opioids for treating breakthrough cancer pain. Breakthrough pain, a temporary exacerbation of pain that may occur spontaneously or be triggered by a specific factor, is common and debilitating for many cancer patients (7), even for those who are well managed with opioids for their background cancer pain (8). The sudden and unpredictable nature of breakthrough pain onset requires a fast-acting approach to pain. The paper reviews several options and provides a practical guide for the use of fast-acting opioids. They also discuss special consideration for various issues, including mucositis, elderly, and polypharmacy issues. Clearly, the safety issues are of significant concern, and the authors discuss the role of education and specific considerations for daily practice.

Treatments of cancer pain are not limited to interventional or pharmacotherapeutic approaches. A wide range of behavioral and complementary methods are available. Acupuncture is one of the most popular modalities for a range of physical ailments. For non-cancer chronic pain, a large volume of trials evaluating the benefits of acupuncture for pain management exists, demonstrating promising results (9). In this special issue, Yan et al. extend this line of investigation into the management of very challenging pain conditions, cancer-induced bone pain, and present a meta-analytic systematic review. Their thorough analyses included 13 studies with a total of over 1,000 patients, generally yielding the favorable benefit of acupuncture for cancer-induced bone pain. The remarkable aspect of this paper is that it also included the effects of acupuncture on other pain-related outcomes, such as analgesic onset time and duration, quality of life, and safety. Unfortunately, they have concluded that the quality of the studies was mostly poor and encouraged further trials. However, the systematic analyses in this paper provide invaluable information on the use of acupuncture for this very difficult pain condition.

The articles in this issue represent some of the ongoing innovations at the transdisciplinary levels, from the molecular and genotype focus to the clinical management of cancer pain. It also reflects the diversity of cancer pain. Cancer pain may come directly from the tumor burden, or it can also arise in response to treatment. It may come in the form of chronic pain or unpredictable episodes of breakthrough pain. Cancer pain management has come a long way and continues to evolve. The issue highlights some pressing needs, providing much-needed overviews of scientific and clinical gems in cancer pain research.
